# Supplementary material for: A Novel Simulation Method of Micro-Topography for Grinding Surface
Source: Materials (Basel). 2021 Sep 7;14(18):5128. doi: 10.3390/ma14185128 (PMC8470869; doi:10.3390/ma14185128)
Supplement: Supplementary file 1 [file materials-14-05128-s001.zip › materials-1323741-SI.pdf]

materials

A Novel Simulation Method of Micro–Topography for Grinding Surface

Qi An *, Shuangfu Suo and Yuzhu Bai

Department of Mechanical Engineering, Tsinghua University, Beijing 100084, China; sfsuo@tsinghua.edu.cn (S.S.); baiyuzhu403@163.com (Y.B.)
* Correspondence: thaq@mail.tsinghua.edu.cn

Citation: An, Q.; Suo, S.; Bai, Y. A Novel Simulation Method of Micro–Topography for Grinding Surface. *Materials* **2021**, *14*, 5128. https://doi.org/10.3390/ma14185128

Academic Editor: Albert Verdaguer

Received: 17 July 2021
Accepted: 2 September 2021
Published: 7 September 2021

Publisher's Note: MDPI stays neutral with regard to jurisdictional claims in published maps and institutional affiliations.

Copyright: © 2021 by the authors. Licensee MDPI, Basel, Switzerland. This article is an open access article distributed under the terms and conditions of the Creative Commons Attribution (CC BY) license (http://creativecommons.org/licenses/by/4.0/).

Table S1. Roughness parameters of measured topography and simulation topography.

v_w/(m/s)		*Sa*/(μm)			*Sq*/(μm)			*Ssk*			*Sku*		
		**Value**	**Mean Value**	**Standard Deviation**	**Value**	**Mean Value**	**Standard Deviation**	**Value**	**Mean Value**	**Standard Deviation**	**Value**	**Mean Value**	**Standard Deviation**
1	Measured topography	0.342			0.462			−0.206			2.365		
		0.337	0.344	0.008185	0.475	0.465	0.008888	−0.211	−0.203	0.009849	2.455	2.375	0.075498
		0.353			0.458			−0.192			2.305		
	Simulation topography	0.352			0.463			−0.206			2.491		
		0.343	0.350	0.006245	0.469	0.463	0.006	−0.191	−0.199	0.00755	2.421	2.471	0.043589
		0.355			0.457			−0.2			2.501		
5	Measured topography	0.391			0.526			−0.185			2.106		
		0.407	0.403	0.010583	0.532	0.523	0.010817	−0.205	−0.195	0.01	2.206	2.136	0.060828
		0.411			0.511			−0.195			2.096		
	Simulation topography	0.402			0.536			−0.194			2.252		
		0.412	0.410	0.007211	0.528	0.535	0.006557	−0.185	−0.187	0.006245	2.182	2.242	0.055678
		0.416			0.541			−0.182			2.292		
10	Measured topography	0.445			0.58			−0.196			2.81		
		0.435	0.445	0.01	0.56	0.569	0.010149	−0.177	−0.184	0.01044	2.78	2.760	0.06245
		0.455			0.567			−0.179			2.69		
	Simulation topography	0.44			0.571			−0.18			2.665		
		0.445	0.439	0.006557	0.562	0.570	0.00755	−0.172	−0.179	0.006557	2.655	2.685	0.043589
		0.432			0.577			−0.185			2.735		
15	Measured topography	0.515			0.622			−0.181			2.608		
		0.502	0.513	0.010149	0.645	0.632	0.01179	−0.174	−0.183	0.010149	2.608	2.578	0.051962
		0.522			0.629			−0.194			2.518		
	Simulation topography	0.505			0.653			−0.19			2.595		
		0.496	0.504	0.00755	0.646	0.645	0.008544	−0.2	−0.192	0.007211	2.645	2.605	0.036056
		0.511			0.636			−0.186			2.575		

Table S2. Relative error of the roughness parameters between measured topography and simulation topography.

v_w/(m/s)		*Sa*/(μm)	*Sq*/(μm)	*Ssk*	*Sku*
1	Measured topography	0.344	0.465	−0.203	2.375
	Simulation topography	0.35	0.463	−0.199	2.471
	Relative error/%	−1.71%	0.43%	2.01%	−3.89%
5	Measured topography	0.403	0.523	−0.195	2.136
	Simulation topography	0.41	0.535	−0.187	2.242
	Relative error/%	−1.71%	−2.24%	4.28%	−4.73%
10	Measured topography	0.445	0.569	−0.184	2.76
	Simulation topography	0.439	0.57	−0.179	2.685
	Relative error/%	1.37%	−0.18%	2.79%	2.79%
15	Measured topography	0.513	0.632	−0.183	2.578
	Simulation topography	0.504	0.645	−0.192	2.605
	Relative error/%	1.79%	−2.02%	−4.69%	−1.04%
